# Exogenous melatonin alleviates copper stress in apple rootstock M9T337 by regulating the antioxidant system and carbon–nitrogen metabolism

**DOI:** 10.3389/fpls.2026.1793846

**Published:** 2026-03-18

**Authors:** Fen Wang, Jufanhang Zhang, Xue Hao, Ling Peng, Shuo Ding, Chenyu Zhao, Longzhe Liu, Xiang Ji, Xuehui Zhao, Maoxiang Sun, Ruirui Xu

**Affiliations:** 1 Key Laboratory of Biochemistry and Molecular Biology in University of Shandong Province, College of Advanced Agriculture and Life Sciences, Weifang University, Weifang, Shandong, China; 2 College of Horticulture and Landscape Architecture, Yangzhou University, Yangzhou, Jiangsu, China; 3 Shandong Key Laboratory of Eco-Environmental Science for Yellow River Delta, Shandong University of Aeronautics, Binzhou, Shandong, China

**Keywords:** apple, copper stress, melatonin, antioxidant, carbon–nitrogen metabolism

## Abstract

Excess copper (Cu) in the soil has become a key environmental stress factor constraining the high quality and yield of apple. Melatonin (MT) has significant potential in regulating plant stress resistance. This study used apple rootstock M9T337 in a hydroponic experiment with four treatments: control (CK), 100 μmol·L^−1^ MT (MT), 30 μmol·L^−1^ CuSO_4_ (Cu), and Cu+MT. Isotopic labeling, noninvasive micro-test technology, and quantitative real-time PCR (qRT-PCR) were used to investigate the mechanisms by which MT affects the growth, antioxidant system, and carbon–nitrogen metabolism of apple rootstock under Cu stress. Copper stress inhibited seedling growth, deteriorated root morphology, decreased root activity, and led to carbon-nitrogen metabolism imbalance. Exogenous MT application alleviated these inhibitory effects. Compared to Cu treatment, Cu+MT treatment decreased H_2_O_2_ and malondialdehyde content and increased seedlings biomass, leaf superoxide dismutase, peroxidase, catalase, and ascorbate peroxidase activities, net photosynthetic rate (P_n_) and rubisco activity, and nitrate reductase, nitrite reductase, glutamine synthetase, and glutamate synthase activities and root tip NO_3_^−^ flux transitions from efflux to uptake. Moreover, Cu+MT treatment increased ^13^C and ^15^N accumulation compared to Cu treatment. qRT-PCR showed that MT upregulated the expression of MT synthesis genes (e.g., *MdTDC*), copper detoxification genes (e.g., *MdCCS*), carbon metabolism genes (e.g., *MdSUSY1*), and nitrogen metabolism genes (e.g., *MdNRT1.1*), while downregulating the expression of copper absorption genes (e.g., *MdCOPT2*) and copper transport genes (e.g., *MdYSL3*). MT effectively alleviates Cu stress inhibition in apple rootstock by enhancing the antioxidant capacity, regulating key enzyme activities and gene expression in carbon–nitrogen metabolism, and optimizing the allocation of photosynthetic products and nitrogen. These results provide a theoretical basis for managing Cu pollution in apple orchards.

## 1 Introduction

Apple is an important economic crop cultivated worldwide. China is a major apple producer, accounting for over 40% of the world’s production area and yield (Chen et al., 2025). Wang et al. (2015) found that the soil Cu content in apple orchards increased with orchard age, exceeding the Cu content in orchards not treated with Cu agents long term. Specifically, the soil Cu content in apple orchards in the Jiaodong Peninsula of China was 3.5 times higher than that in local agricultural soil. In Liaodong Peninsula orchards, 5.56% of soil samples exceeded the permissible Cu standard (150 mg kg^−1^), showing a maximum concentration of 229 mg kg^−1^ (Fu et al., 2020). An abnormally high soil Cu content inhibits the root growth of apple rootstocks, weakening their ability to absorb water and nutrients and indirectly interfering with carbon and nitrogen metabolism. Cu stress significantly inhibits root elongation, lateral root development, and root activity in apple rootstocks, decreasing their absorption capacity for mineral elements, such as nitrogen, which affects nitrogen metabolism (Wan et al., 2019). Cu stress also alters the photosynthetic characteristics of apple rootstock leaves and reduces photosynthetic efficiency and the accumulation of photosynthetic products, negatively impacting carbon metabolism (Li et al., 2024; Wang et al., 2025). Alleviating the damage caused by Cu stress in apples through exogenous substances is crucial for improving apple quality, production efficiency, and public health.

Melatonin (MT), a multifunctional signaling molecule widely present in plants, has recently gained attention for its potential to enhance plant stress resistance (Arnao and Hernández-Ruiz, 2019; Huang et al., 2023; Ruan et al., 2025). It regulates key stages of plant growth and development, such as root growth, flowering, and fruiting, and plays a central role in biotic and abiotic stress responses (Arnao and Hernández-Ruiz, 2021). MT has a strong antioxidant capacity, effectively scavenging excess reactive oxygen species (ROS) and reactive nitrogen species (RNS) produced under stress, mitigating oxidative damage, and maintaining normal cellular physiological functions. Under abiotic stressors, such as heavy metals, drought, salinity, high temperatures, and low temperatures, exogenous MT application significantly enhances plant stress resistance, growth, and development and reduces oxidative damage and ion toxicity (Back et al., 2016; Liu et al., 2025b; Ruan et al., 2025). MT improved plant drought resistance by regulating osmotic adjustment substances, the antioxidant enzyme activity, and stomatal conductance (Liang et al., 2018). Exogenous MT significantly alleviated the inhibitory effects of Cu on tomato growth, enhanced plant tolerance to Cu ions, increased antioxidant enzyme activities and root activity, and reduced the proline and malondialdehyde contents (Zhang et al., 2022).

MT shows great potential for regulating carbon and nitrogen metabolism. MT can regulate plant hormone signaling pathways, affect related enzyme activities and gene expression, promote nitrogen absorption and assimilation, and optimize carbohydrate allocation and utilization (Erdal, 2019). It can also increase photosynthetic pigment and enzyme activities, providing the material basis for carbon and nitrogen metabolism (Yang et al., 2022). However, the mechanism by which MT responds to Cu stress in apple rootstocks, especially its impact on carbon-nitrogen metabolism, has not yet been fully elucidated.

This experiment used tissue-cultured seedlings of the commonly used apple dwarfing rootstock M9T337. Using hydroponics, isotopic labeling, and noninvasive micro-test technology (NMT), the effects of MT on the growth, MT synthesis, antioxidant enzyme activities, and carbon–nitrogen metabolism of apple rootstock seedlings under Cu stress were investigated. The results of this study are significant for improving apple production quality and efficiency.

## 2 Materials and methods

### 2.1 Plant materials and treatments

The experiment was conducted from March to June 2024 at Weifang University in Weifang, Shandong Province, China. One-year-old tissue-cultured seedlings of apple dwarfing rootstock M9T337 were cultured in a light incubator maintained at 28°C during the day and 18°C at night, with a humidity level of 55%. Rootstocks with consistent growth (approximately 10 cm high) were planted in a hydroponic box with 10 holes in each foam board. A single rootstock was placed in each hole, and 6 L of nutrient solution was added to each box (hydroponic box dimensions: 40 cm length, 28 cm width, 15 cm height). To gradually acclimate the rootstocks to the nutrient solution, they were initially placed in 1/2 Hoagland’s nutrient solution (containing 2.5 mM Ca(NO_3_)_2_, 2.5 mM KNO_3_, 0.5 mM KH_2_PO_4_, 1 mM MgSO_4_, 0.1 mM Fe-EDTA, 20 μM H_3_BO_3_, 4.5 μM MnCl_2_, 0.4 μM ZnSO_4_, and 0.2 μM CuSO_4_) for 5 days. They were then transferred to full-strength Hoagland’s nutrient solution for an additional 5 days. The nutrient solution was replaced every 5 days. After 15 days, the rootstocks with consistent growth were selected for the trial.

In the trial, the following treatments were applied to the seedlings: control (CK), 100 μmol·L^−1^ MT, 30 μmol·L^−1^ CuSO_4_·5H_2_O (Cu), and 100 μmol·L^−1^ MT + 30 μmol·L^−1^ CuSO_4_·5H_2_O (Cu+MT). The MT concentration was selected based on Sun et al. (2024), and the Cu concentration was based on pre-trial results that showed that this concentration could induce Cu toxicity in apple. Both MT and Cu^2+^ were prepared fresh. For Cu+MT treatment, CuSO_4_ was added first, followed by MT 2 h later. Each treatment was applied to 3 independent hydroponic boxes, with 10 uniformly grown seedlings per box. The experimental nutrient solutions were formulated using deionized water and were replaced every 5 days during the experiment, with regular aeration for 12 h each day. The pH of the nutrient solution was monitored daily using a pH meter (PHS-3E, LEICI, China) and maintained at 6.0 ± 0.1 using either H_3_PO_4_ or NaOH. Samples were collected after 30 days of treatment to measure the physiological indicators and gene expression levels.

For ^15^N labeling, 0.5 g Ca(^15^NO_3_)_2_ (10.22% abundance) was added to each box with nutrient solution. ^13^C labeling was performed after 27 days of treatment. The labeling source was Ba^13^CO_3_ (^13^C abundance 98%, dosage 1 g per box). The seedlings, a beaker containing Ba^13^CO_3_, a small fan, and ice packs were placed in a self-made transparent film labeling chamber. Labeling started at 09:00 with the fan turned on, and the chamber was sealed. To maintain the CO_2_ concentration, 1 mL of 1 mol·L^−1^ HCl was injected into the beaker containing Ba^13^CO_3_ every 0.5 h, and labeling lasted for 4 h. Ice packs were placed at the bottom of the chamber during labeling to maintain a temperature between 28 and 37 °C. Another 3 seedlings were selected as controls for natural ^13^C abundance. Destructive sampling was performed 3 days after labeling (30th day of the experimental treatment) to determine the ^13^C and ^15^N abundance (Xu et al., 2023).

### 2.2 Determination of the biomass, root system indicators, and photosynthetic parameters

Plant samples were washed and blotted dry. The fresh weights of the roots, stems, and leaves were then measured separately. Root system characteristics (root length, number of root tips, root volume, and root surface area) were analyzed using the WinRhizo system (version 2012b, Montreal, Canada). Root activity was determined using the triphenyl tetrazolium chloride (TTC) reduction method (Liu et al., 2025). Measure the photosynthetic parameters (P_n_, net photosynthetic rate; Gs, stomatal conductance; Ci, intercellular CO_2_ concentration) of the leaves using the Li-6400 portable photosynthesis system (LI-COR Inc., USA).

### 2.3 Determination of melatonin content and copper content

The MT content was determined according to the method described by Cao et al. (2023). A 0.5-g sample was ground into powder in liquid nitrogen, and methanol was added. After ultrasonication and centrifugation, the supernatant was passed through a 0.22-μm organic filter membrane. The MT content was determined in the leaves using high-performance liquid chromatography (HPLC). The sample was digested using H_2_SO_4_-H_2_O_2_, and the Cu content in the roots, stems, and leaves was measured using an atomic absorption spectrophotometer.

### 2.4 Determination of the antioxidant enzyme activities and reactive oxygen species content

The superoxide dismutase (SOD) activity was determined based on its ability to inhibit the photochemical reduction of nitroblue tetrazolium. The peroxidase (POD) activity was determined by monitoring the absorbance change at 470 nm due to the oxidation of guaiacol to tetraguaiacol. The catalase (CAT) activity was measured using ultraviolet spectrophotometry at 240 nm. The ascorbate peroxidase (APX) activity was measured using the NADH method. The H_2_O_2_ content was determined as described by (Guan et al., 2025). H_2_O_2_ was extracted from samples with pre-cooled acetone and detected by monitoring the absorbance of the titanium peroxide complex at 412 nm. The determination of malondialdehyde (MDA) content refers to (Hadian-Deljou et al., 2020).

### 2.5 Determination of sugar metabolism enzymes and products

The sucrose phosphate synthase (SPS) and sucrose synthase (SS) activities were quantified by measuring the amount of sucrose generated from uridine diphosphate glucose. The rubisco activity in leaves was determined using the NADH-coupled spectrophotometric method. The content of sucrose (Suc), fructose (Fru), and glucose (Glu) were determined as described by Aslam et al. (2019).

### 2.6 Measurement of NO_3_^−^ flux

The NO_3_^−^ flux at the surface of seedling root tips was measured using an NMT system (NMT 100 Series, MA, USA). Measurements were taken in the area 300 μm away from the root apex. Each selected root was tested for 10 min, and there were 7 replicates per treatment. After the test, data were analyzed using MageFlux (imFluxes V2.0, MA, USA).

### 2.7 Determination of key nitrogen assimilation enzymes and products

The nitrate reductase (NR) activity was determined using the sulfanilamide colorimetric method (Sun et al., 2024). The nitrite reductase (NiR), glutamine synthetase (GS), and glutamate synthase (GOGAT) activities were measured using plant enzyme activity assay kits (Suzhou Comin Biotechnology Co., Ltd., Jiangsu, China). The amino acid content was determined using liquid chromatography-mass spectrometry (LC-MS). The soluble protein content was determined using the Coomassie Brilliant Blue G-250 staining method (Liu et al., 2025).

### 2.8 Isotope analysis

For isotope analysis, each treatment was set with three biological replicates (i.e., from three independent hydroponic boxes). Samples were dried at 80°C to a constant weight, crushed, and passed through a 0.25-mm sieve. The abundance of ^13^C and ^15^N was measured using an isotope ratio mass spectrometer (Thermo Fisher Scientific Inc., Waltham, MA, USA), and there were 3 replicates per treatment. Data related to ^13^C and ^15^N were calculated according to the formula given by Wang et al. (2021).

### 2.9 Quantitative real-time PCR detection of genes

The following genes were selected for analysis by qRT-PCR: nitrogen assimilation-related genes, such as nitrate reductase (*MdNR*), nitrous reductase (*MdNiR*), glutamine synthetase (*MdGS*), Fd-dependent glutamate synthase (*MdFd*-*GOGAT*), and NADH-dependent glutamate synthase (*MdNADH*-*GOGAT*); nitrate transporters, such as nitrate transporters (*MdNRT1.1*, *MdNRT1.2*, *MdNRT2.1*, *MdNRT2.4*); carbon assimilation-related genes, such as sucrose synthase (*MdSUSY1*), sucrose transporter (*MdSUT1*), sucrose phosphate synthase (*MdSPS1*), and hexokinase (*MdHK6*); copper uptake genes (*MdCOPT1*, *MdCOPT2*, *MdCOPT6*, *MdZIP2*, and *MdZIP4*), copper transport genes (*MdYSL3*, *MdHMA5*), and copper detoxification genes (*MdABCC2*, *MdCSD1*, and *MdCCS*); and MT synthesis-related genes, such as tryptophan decarboxylase (*MdTDC*), tryptamine 5-hydroxylase (*MdT5H*), serotonin N-acetyltransferase (*MdSNAT*), and N-acetylserotonin O-methyltransferase (*MdASMT*). Total RNA was extracted using an RNAprep Pure Plant Kit (Tiangen Biotech Co., Ltd., Beijing, China). RNA was reverse transcribed into cDNA. qRT-PCR was performed in a reaction mixture containing 10 μL of Green qPCR SuperMix, 1 μL of cDNA, 2 μL of primers, and 7 μL of ddH_2_O (An et al., 2022). The relative gene expression levels were calculated using the 2^–ΔΔCT method, and the *MdActin* gene was used as the internal reference (Zhang et al., 2023). qRT-PCR experiments were performed with three technical replicates and three biological replicates. The primer sequences used for qRT-PCR are listed in **Supplementary Table 1**.

### 2.10 Data analysis

Statistical analysis was performed using IBM SPSS Statistics software (version 19.0). Differences between means were determined using the least significant difference (LSD) test, with a *P*-value < 0.05 considered significant. Graphs were generated using GraphPad Prism software (version 10.3), OmicStudio tools (https://www.omicstudio.cn/tool), and Wei Sheng Xin platform (https://www.bioinformatics.com.cn).

## 3 Results

### 3.1 Seedling growth indicators

**Figure 1:**
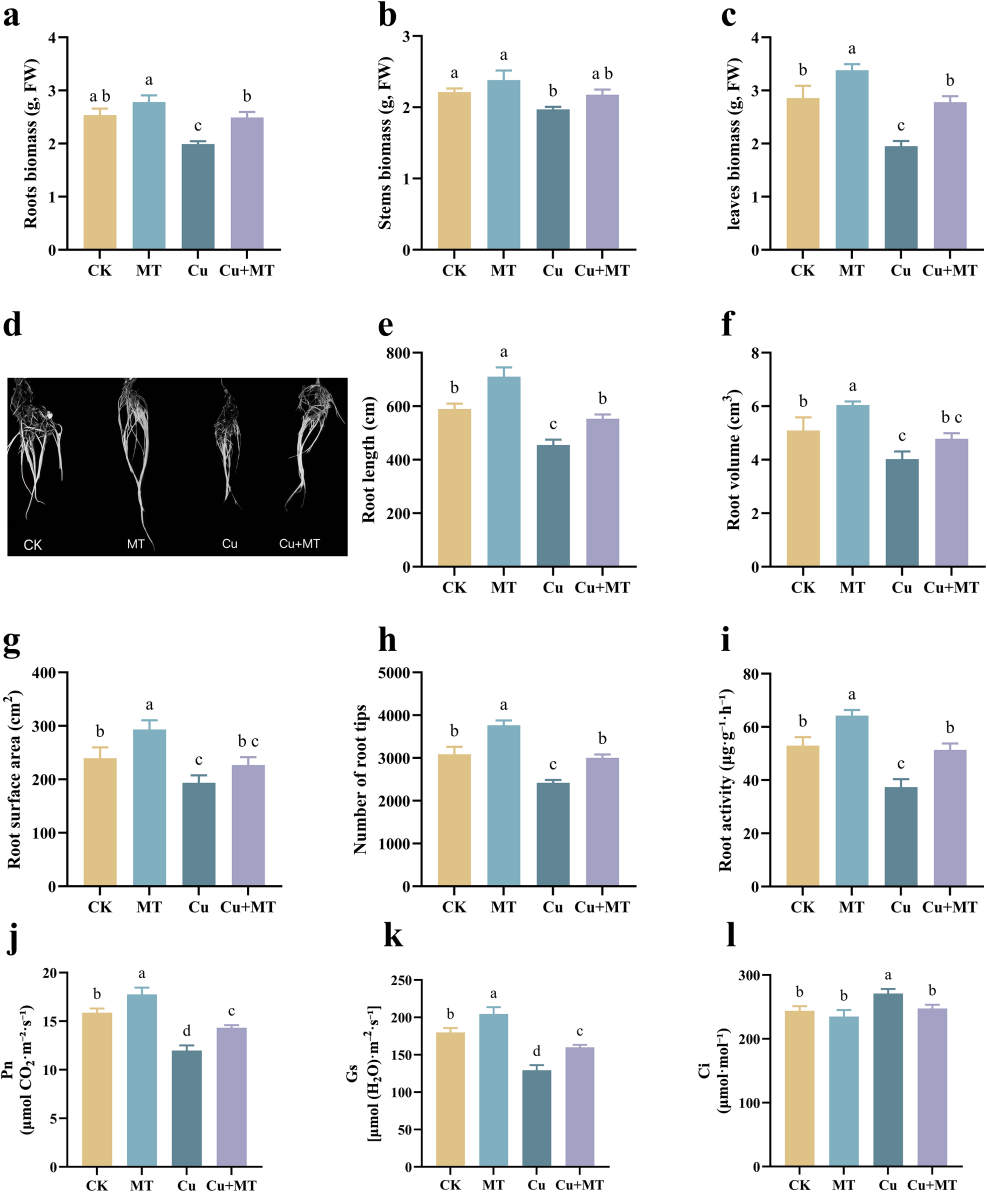
Effects of different treatments on biomass and root system characteristics of M9T337 rootstock. Roots biomass **(a)**, stems biomass **(b)**, leaves biomass **(c)**, root morphology **(d)**, root length **(e)**, root volume **(f)**, root surface area **(g)**, number of root tips **(h)**, root activity **(i)**, P_n_ **(j)**, Gs **(k)**, and Ci **(l)**. Different letters indicate statistically significant differences among treatments (*P* < 0.05). The data are presented as mean ± standard deviation (n = 3).

As shown in **Figure 1**, Cu stress significantly reduced the root, stem, and leaf biomass of the seedlings (*P* < 0.05) by 21.46, 11.26, and 31.60%, respectively, compared to CK. However, Cu+MT treatment significantly alleviated the inhibitory effects of Cu stress on root, stem, and leaf growth, increasing them by 25.13, 10.66, and 42.56%, respectively, compared to Cu treatment. Analysis of root system characteristics revealed that Cu treatment significantly inhibited seedling root length, root volume, root surface area, and the number of root tips compared to CK (**Figures 1d–h**). MT treatment significantly improved seedling root morphology indicators, and Cu+MT treatment showed that MT addition significantly improved root growth inhibition caused by Cu stress. Cu+MT treatment increased the root length, number of root tips, surface area, and volume by 21.52, 24.07, 17.21, and 18.66%, respectively, compared to Cu treatment. Root activity showed a similar trend (**Figure 1**). This indicates that MT addition alleviates root growth inhibition in apple seedlings caused by Cu stress.

As shown in **Figure 1j**, different treatments significantly affected the P_n_ of M9T337 seedlings. Compared to CK, MT treatment significantly increased the P_n_ of apple seedlings (P < 0.05). Cu+MT treatment showed that MT significantly alleviated the inhibitory effect of Cu on P_n_, increasing it by 19.62%. This suggests that under Cu stress conditions, appropriate MT application effectively enhanced the P_n_ of M9T337 seedlings. Compared to CK, Cu stress significantly inhibited the stomatal conductance (Gs) of seedlings (decreased by 28.16%). Compared to Cu treatment, the addition of MT (Cu+MT treatment) improved Gs (P < 0.05). Compared to CK, Cu stress increased the intercellular CO_2_ concentration (Ci), and the addition of MT (Cu+MT treatment) could reduce Ci to the control level.

### 3.2 Antioxidant enzyme activities, and ROS content

**Figure 2:**
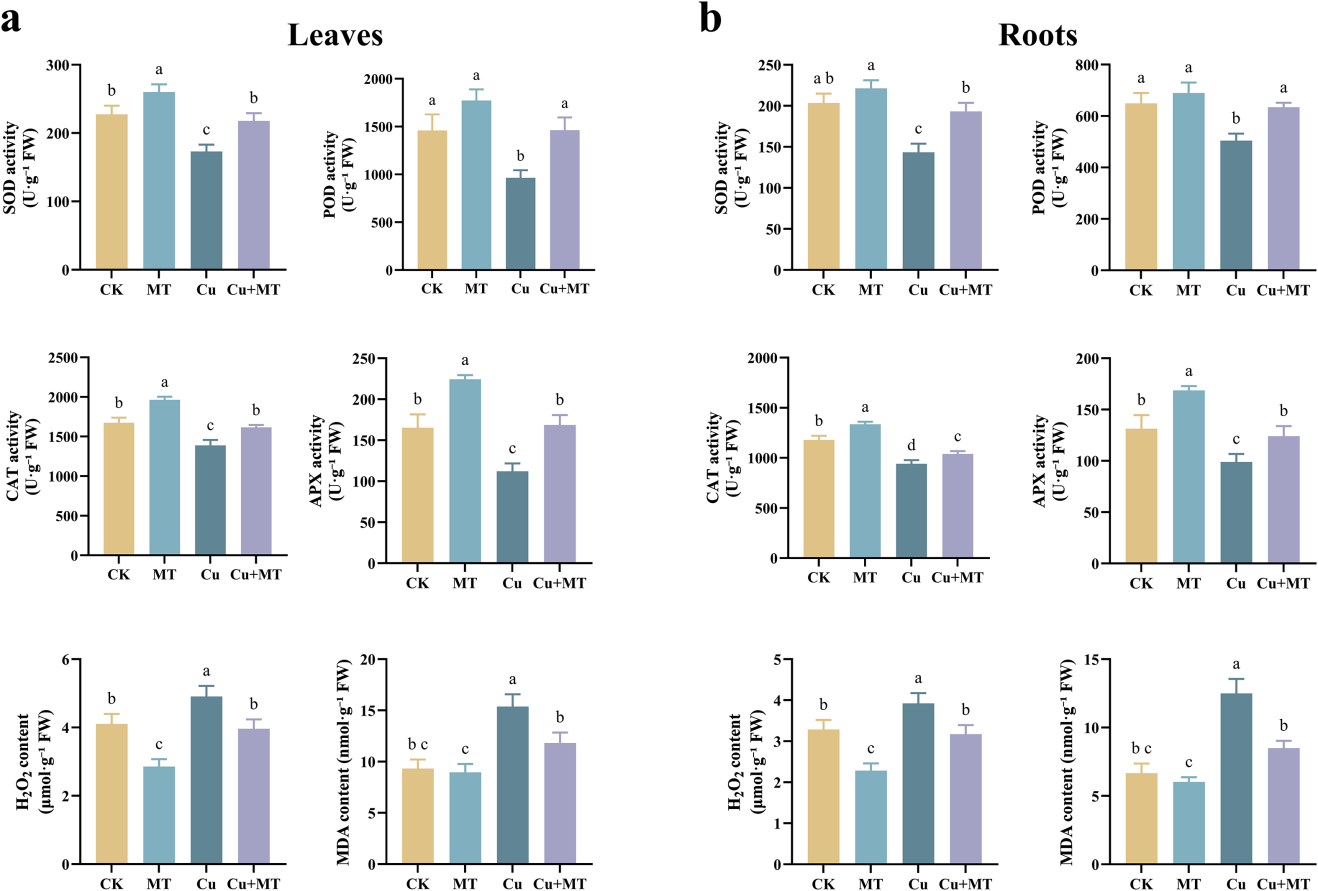
Effects of different treatments on the Leaves antioxidant enzyme activities and ROS content **(a)** and roots antioxidant enzyme activities and ROS content **(b)**. Different letters indicate statistically significant differences among treatments (*P* < 0.05). The data are presented as mean ± standard deviation (n = 3).

As shown in **Figure 2**, different treatments affected the antioxidant enzyme activities in M9T337 seedling leaves and roots. Compared to CK, MT treatment significantly increased the SOD, POD, CAT, and APX activities in both leaves and roots, indicating that MT promotes antioxidant enzyme synthesis in apple seedlings. Compared to Cu treatment, Cu+MT treatment significantly increased the SOD, POD, CAT, and APX activities in the leaves (*P* < 0.05), by 25.77, 51.79, 16.42, and 50.23%, respectively. Furthermore, compared to Cu treatment, Cu+MT treatment significantly reduced H_2_O_2_ and MDA content. This indicates that MT addition significantly alleviates the inhibition of antioxidant capacity in seedling caused by Cu stress.

### 3.3 Melatonin and Cu content

**Figure 3:**
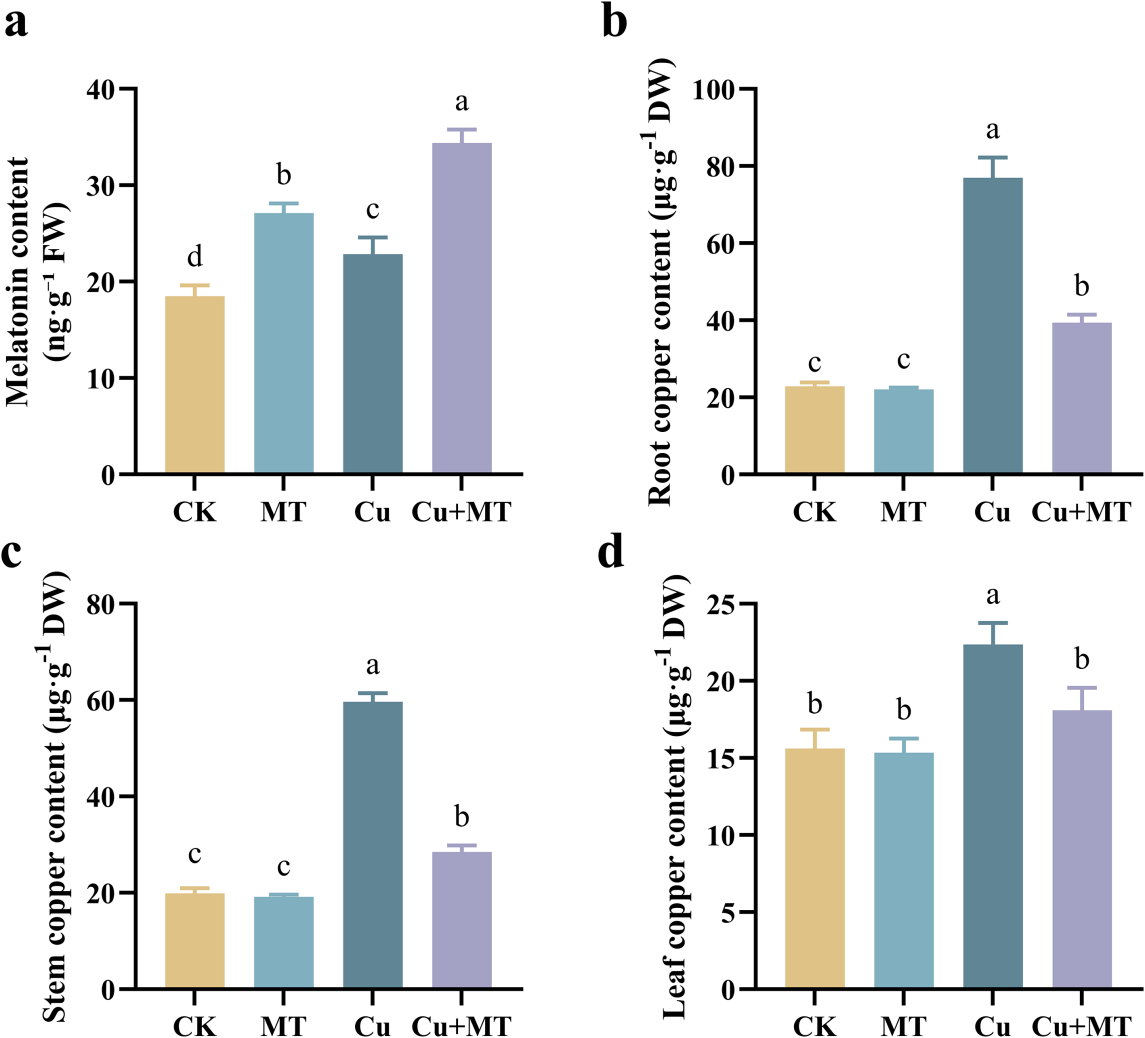
Effects of different treatments on melatonin and copper content in M9T337 rootstocks. Melatonin content **(a)**, root copper content **(b)**, stem copper content **(c)**, and leaf copper content **(d)**. Different letters indicate statistically significant differences among treatments (*P* < 0.05). The data are presented as mean ± standard deviation (n = 3).

MT, Cu, and Cu+MT treatments increased the MT content of seedling leaves to varying degrees (**Figure 3**). Compared to CK, MT, Cu, and Cu+MT treatments increased the MT content by 46.80, 23.76, and 86.16%, respectively. Compared to CK, Cu stress significantly increased the copper content in roots, stems, and leaves by 235.75, 199.96, and 43.19%, respectively. Compared to Cu treatment, the addition of MT (Cu+MT treatment) significantly reduced the copper content in roots, stems, and leaves.

### 3.4 Carbon metabolism and photosynthate allocation

#### 3.4.1 Sugar metabolism

**Figure 4:**
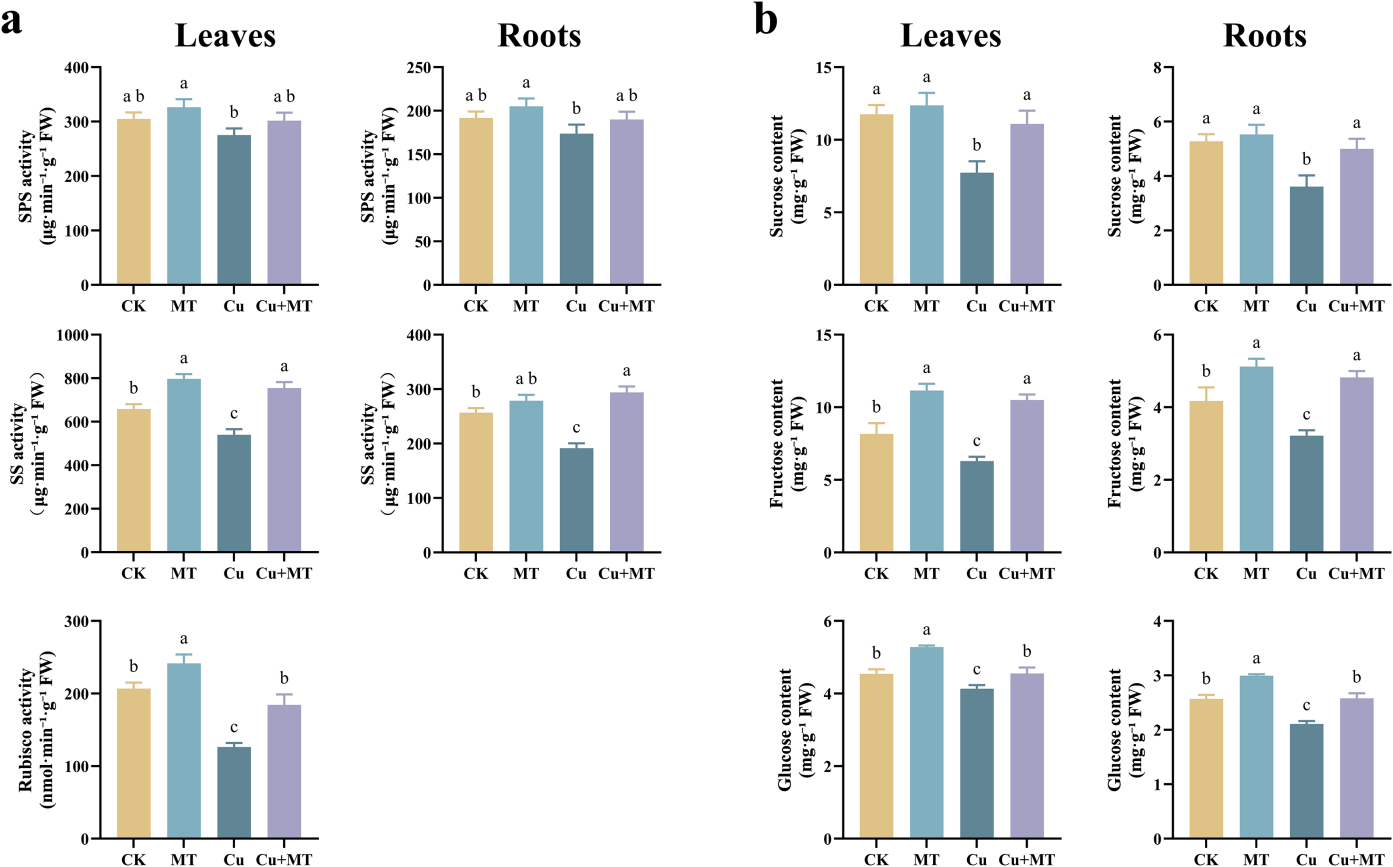
Effects of different treatments on sugar metabolism in M9T337 rootstocks. Sugar metabolism-related enzymes **(a)** and sugar metabolism-related products **(b)**. Different letters indicate statistically significant differences among treatments (*P* < 0.05). The data are presented as mean ± standard deviation (n = 3).

As shown in **Figure 4a**, Cu treatment significantly inhibited the activities of SPS and SS in the leaves and roots of seedlings compared to CK (*P* < 0.05). Cu+MT treatment increased the SPS and SS activities in the leaves by 9.63 and 39.72%, respectively, compared to Cu treatment. This indicates that MT has a significant promoting effect on the activities of these enzymes in apple seedling leaves. MT addition significantly alleviated the inhibition of SPS and SS activities by Cu stress, suggesting that MT effectively mitigates the suppression of sugar metabolism-related enzymes in seedlings under Cu stress. Additionally, MT addition increased the leaf rubisco activity, raising it from 126.42 nmol·min^−1^·g^−1^ under Cu stress to 184.25 nmol·min^−1^·g^−1^.

Different treatments significantly affected the leaf soluble sugar content of the seedlings (**Figure 4b**). The Suc, Glu, and Fru contents in the leaves and roots of the Cu+MT treatment were significantly higher than those in the Cu treatment. Compared to the Cu treatment, the Cu+MT treatment increased the contents of Suc, Glu, and Fru in the leaves by 43.51, 10.00, and 66.56%, and in the roots by 38.53%, 22.22%, and 49.90%, respectively, indicating that MT significantly promotes soluble sugar synthesis in apple seedlings under Cu stress.

#### 3.4.2 ^13^C accumulation and distribution

**Figure 5:**
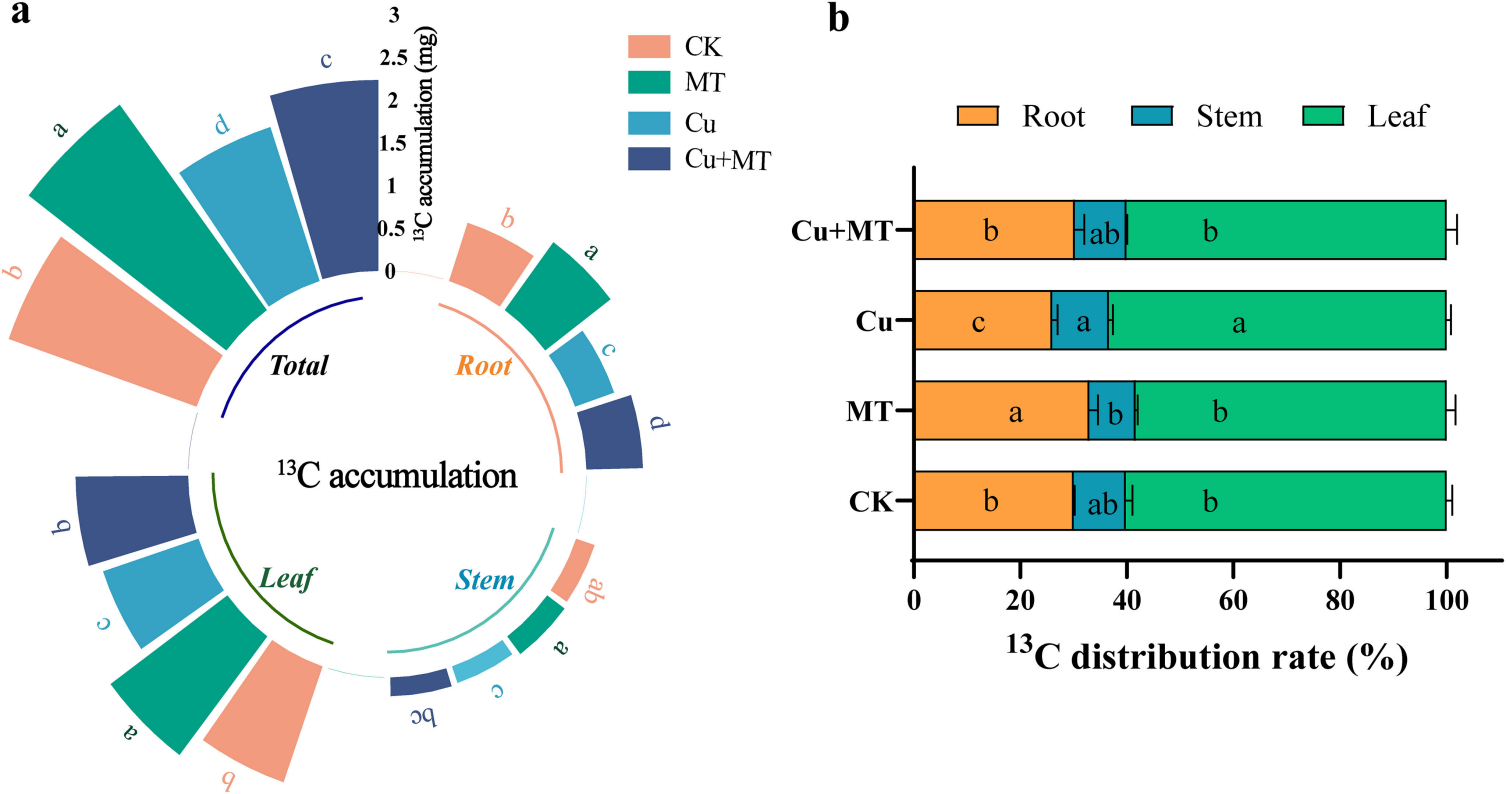
Effects of different treatments on the ^13^C accumulation and distribution rate of M9T337 rootstocks. ^13^C accumulation **(a)** and ^13^C distribution rate **(b)**. Different letters indicate statistically significant differences among treatments (*P* < 0.05). The data are presented as mean ± standard deviation (n = 3).

As shown in **Figure 5**, different treatments had varying effects on ^13^C assimilate accumulation and distribution in M9T337 seedlings. Compared to CK, MT treatment significantly increased ^13^C accumulation in the roots, stems, leaves, and whole plant by 38.03, 11.54, 21.68, and 25.20%, respectively, indicating that MT promotes carbon assimilation product accumulation. MT addition effectively reversed the negative effects of Cu stress. Cu+MT treatment increased ^13^C accumulation in the roots, stems, and leaves by 36.73, 10, and 11.67%, respectively, compared to Cu treatment. Therefore, exogenous MT significantly restored the carbon accumulation capacity of apple rootstock under Cu stress, providing a material basis for maintaining a normal carbon–nitrogen metabolic balance and thereby enhancing its Cu stress tolerance. Analysis of the ^13^C distribution rate revealed that leaves had the highest ^13^C distribution rate, followed by roots, and stems had the lowest. The root ^13^C distribution rate decreased by 13.51% under Cu stress compared to CK, whereas the leaf distribution rate increased by 5.23%. Under Cu+MT treatment, the root ^13^C distribution rate increased by 16.25% compared to Cu treatment, while the leaf distribution rate decreased by 5.19%. This indicates that Cu stress increases the amount of ^13^C assimilates allocated to the leaves, and MT addition promotes the transfer of ^13^C assimilates to the roots.

### 3.5 Nitrogen uptake and metabolism

#### 3.5.1 NO_3_^−^ flux

**Figure 6:**
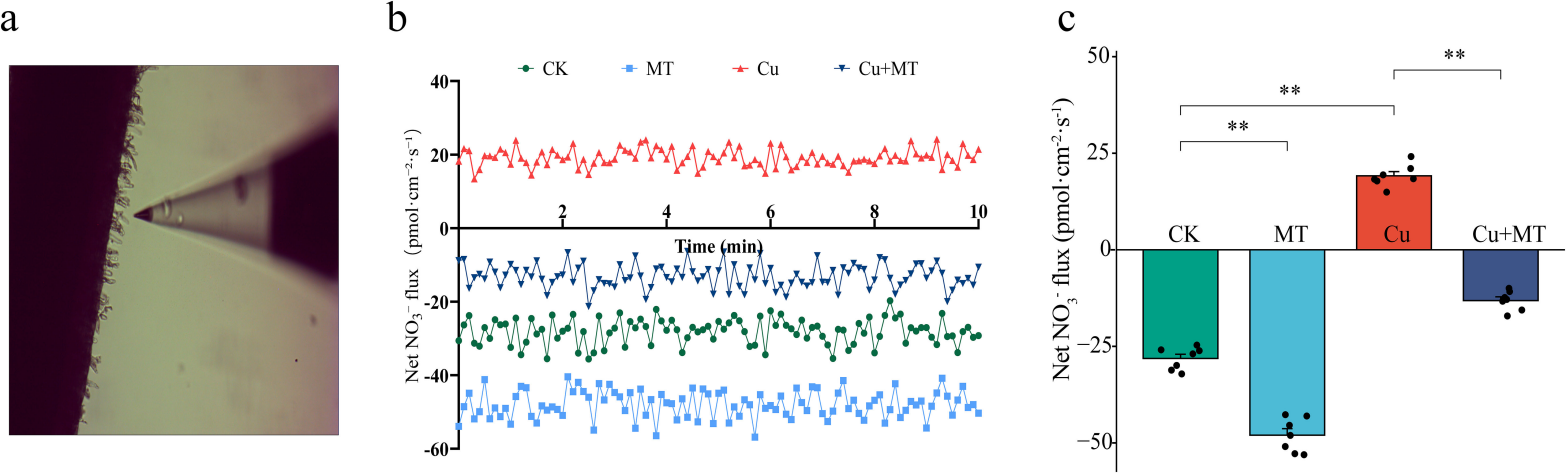
Effects of different treatments on NO_3_^−^ flux in the root tips of M9T337 seedlings. Noninvasive micro-measurement of NO_3_^−^ flow velocity in the root tips of M9T337 seedlings **(a)**, net NO_3_^−^ fluxes in the roots over 10 min **(b)**, and average net NO_3_^−^ flux **(c)**. The data are presented as mean ± standard deviation (n = 7). ** indicates *P* < 0.01.

There were significant differences in the NO_3_^−^ flux rate in the rhizosphere of M9T337 under different treatments (**Figure 6**). Negative values indicate NO_3_^−^ uptake, and positive values indicate NO_3_^−^ efflux. Compared to CK, MT treatment significantly promoted NO_3_^−^ uptake, increasing the average rate from −28.12 to −47.98 pmol·cm^−2^·s^−1^. In contrast, Cu treatment resulted in NO_3_^−^ efflux, with an efflux rate of 19.17 pmol·cm^−2^·s^−1^. In Cu+MT treatment, NO_3_^−^ flux showed uptake behavior, with an average flux rate of approximately −13.13 pmol·cm^−2^·s^−1^. These results indicate that Cu stress is detrimental to NO_3_^−^ uptake and that MT addition under Cu stress conditions favors NO_3_^−^ uptake. This suggests that exogenous MT enhances the Cu tolerance of apple rootstock roots by restoring the ion flux balance at the root tips, thereby improving the nitrate nitrogen uptake rate.

#### 3.5.2 Nitrogen metabolism

**Figure 7:**
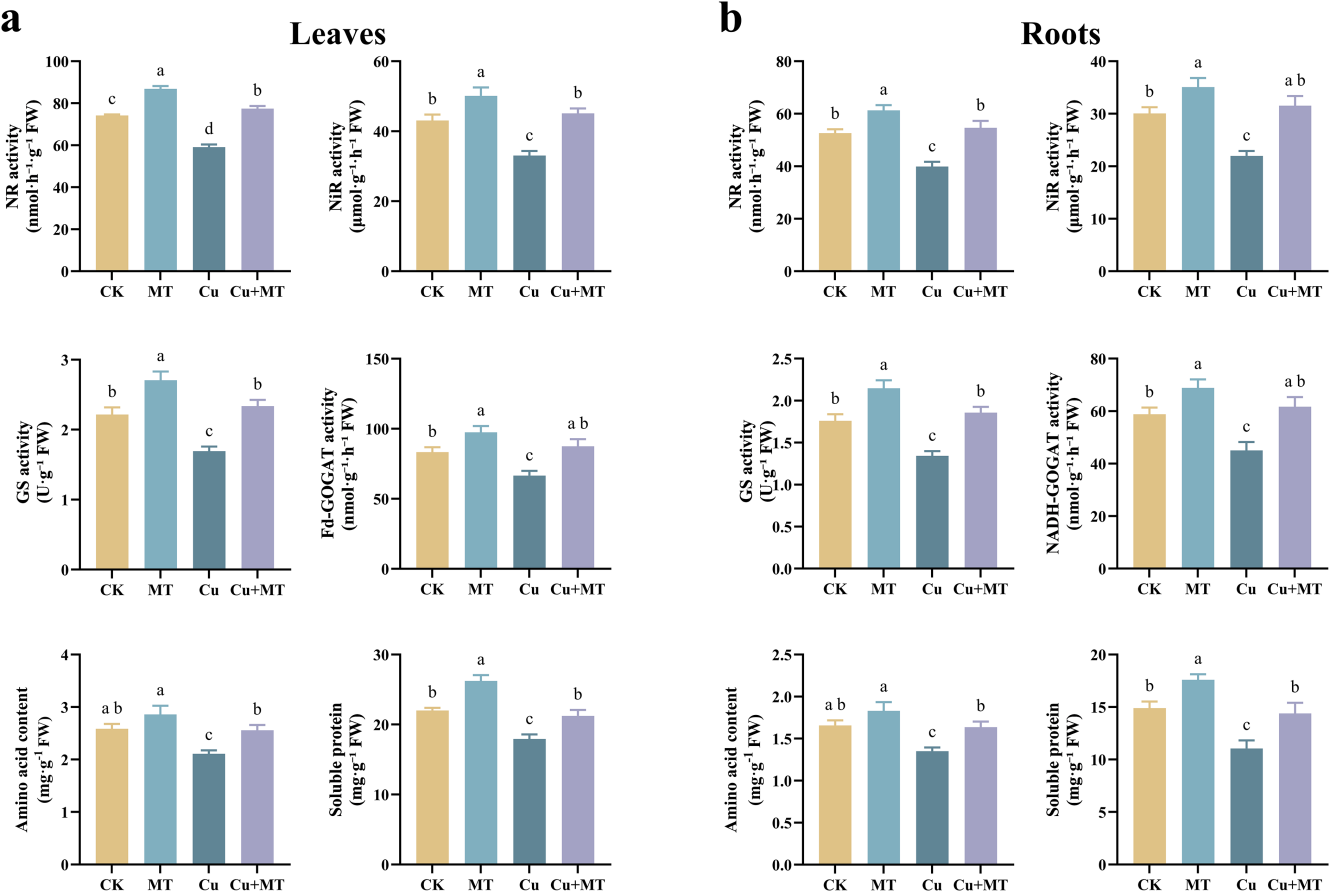
Effects of different treatments on nitrogen metabolism in M9T337 rootstocks. Leaves nitrogen metabolism enzymes and products **(a)** and roots nitrogen metabolism enzymes and products **(b)**. Different letters indicate statistically significant differences among treatments (*P* < 0.05). The data are presented as mean ± standard deviation (n = 3).

As shown in **Figure 7**, different treatments significantly affected the nitrogen metabolism enzyme activities (NR, NiR, GS, and GOGAT) in leaves and roots. Compared to CK, MT treatment significantly increased the NR, NiR, GS, and GOGAT activities in the leaves by 17.01, 16.37, 21.99, and 16.83%, respectively, whereas Cu treatment significantly inhibited the activities of these four enzymes. Cu+MT treatment significantly alleviated the inhibitory effect of Cu stress on nitrogen metabolism enzyme activities (*P* < 0.05). Compared to Cu treatment, the NR, NiR, GS, and GOGAT activities in Cu+MT-treated leaves increased by 23.65, 36.58, 38.46, and 31.23%, respectively. The activity of nitrogen metabolism enzymes in the seedling roots also exhibited the same pattern. This indicates that MT effectively mitigates the negative impact of Cu stress on the nitrogen metabolism process in apple seedlings.

Further investigation into nitrogen metabolism products (amino acid and soluble protein) revealed that MT treatment increased the content of amino acid and soluble protein in the leaves and roots of apple seedlings (**Figure 7**). The amino acid and soluble protein contents were significantly lower under Cu treatment than under CK (*P* < 0.05). Cu+MT treatment effectively reversed the adverse effects of Cu stress, increasing the contents of amino acid and soluble protein by 21.33 and 18.44% in the leaves, respectively, compared to Cu treatment. The results showed that applying an appropriate amount of MT to plants under Cu stress effectively increased the content of amino acid and soluble protein in the leaves and roots of M9T337 seedlings, thereby enhancing their ability to cope with Cu stress.

#### 3.5.3 ^15^N uptake, utilization, and distribution

**Figure 8:**
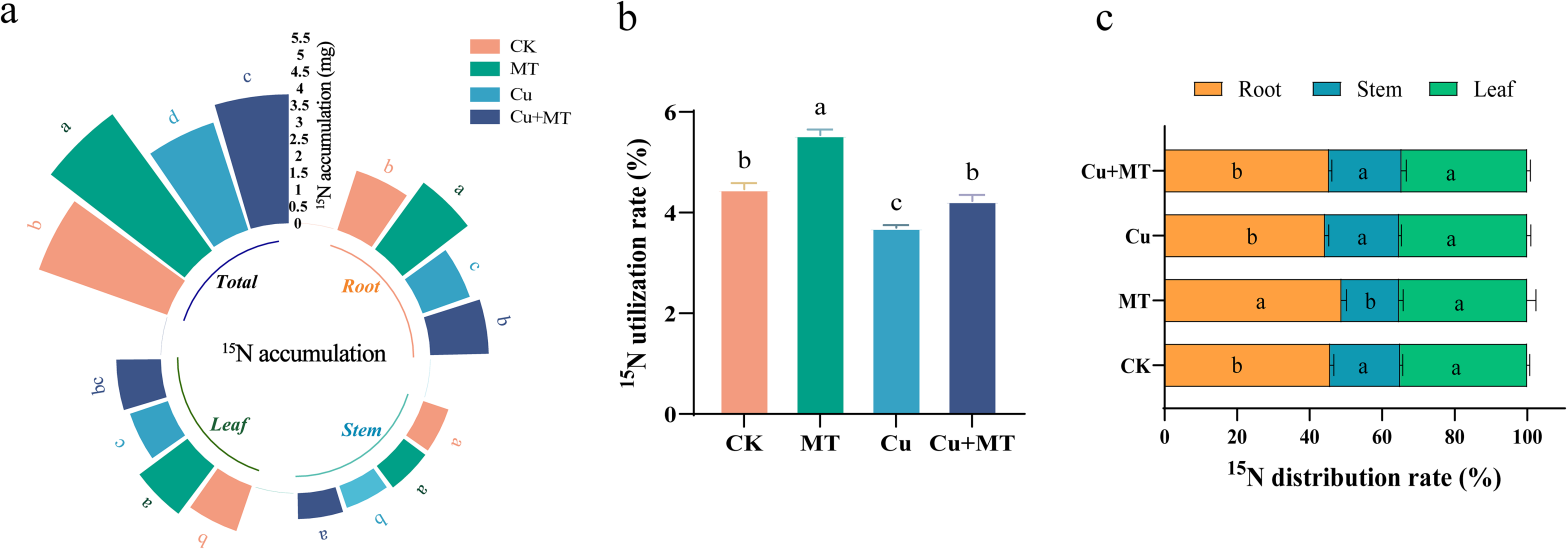
Effects of different treatments on ^15^N uptake, utilization, and distribution in M9T337 rootstocks. ^15^N absorption amount **(a)**, ^15^N utilization rate **(b)**, and ^15^N distribution rate **(c)**. Different letters indicate statistically significant differences among treatments (*P* < 0.05). The data are presented as mean ± standard deviation (n = 3).

As shown in **Figure 8**, different treatments had varying effects on the ^15^N uptake, utilization, and distribution in M9T337 seedlings. Compared to CK, MT treatment significantly increased ^15^N uptake in the roots, leaves, and whole plant by 32.55, 25.38, and 24.11%, respectively (*P* < 0.05), indicating that MT addition promotes nitrogen absorption and accumulation. Cu treatment significantly reduced ^15^N uptake in the roots, stems, leaves, and whole plant. MT addition effectively reversed the negative effects of Cu stress, increasing ^15^N uptake in the roots, stems, and leaves by 16.99, 11.74, and 12.16%, respectively, compared to Cu treatment. Further analysis showed that MT addition improved the seedlings’ ^15^N utilization efficiency, whereas Cu treatment reduced it. The ^15^N utilization rate of MT treatment was 24.11% higher than that of CK, and that of Cu+MT treatment was 14.21% higher than that of Cu treatment. This indicates that MT alleviates the inhibition of nitrogen absorption and utilization caused by Cu stress. Additionally, analysis of the ^15^N distribution rate revealed that roots had the highest ^15^N distribution rate, followed by leaves, and stems had the lowest. Compared to CK, MT treatment increased the root ^15^N distribution rate by 6.86% and decreased the stem distribution rate by 17.89%. Cu treatment decreased the root ^15^N distribution rate by 2.98%.

### 3.6 Gene expression levels

**Figure 9:**
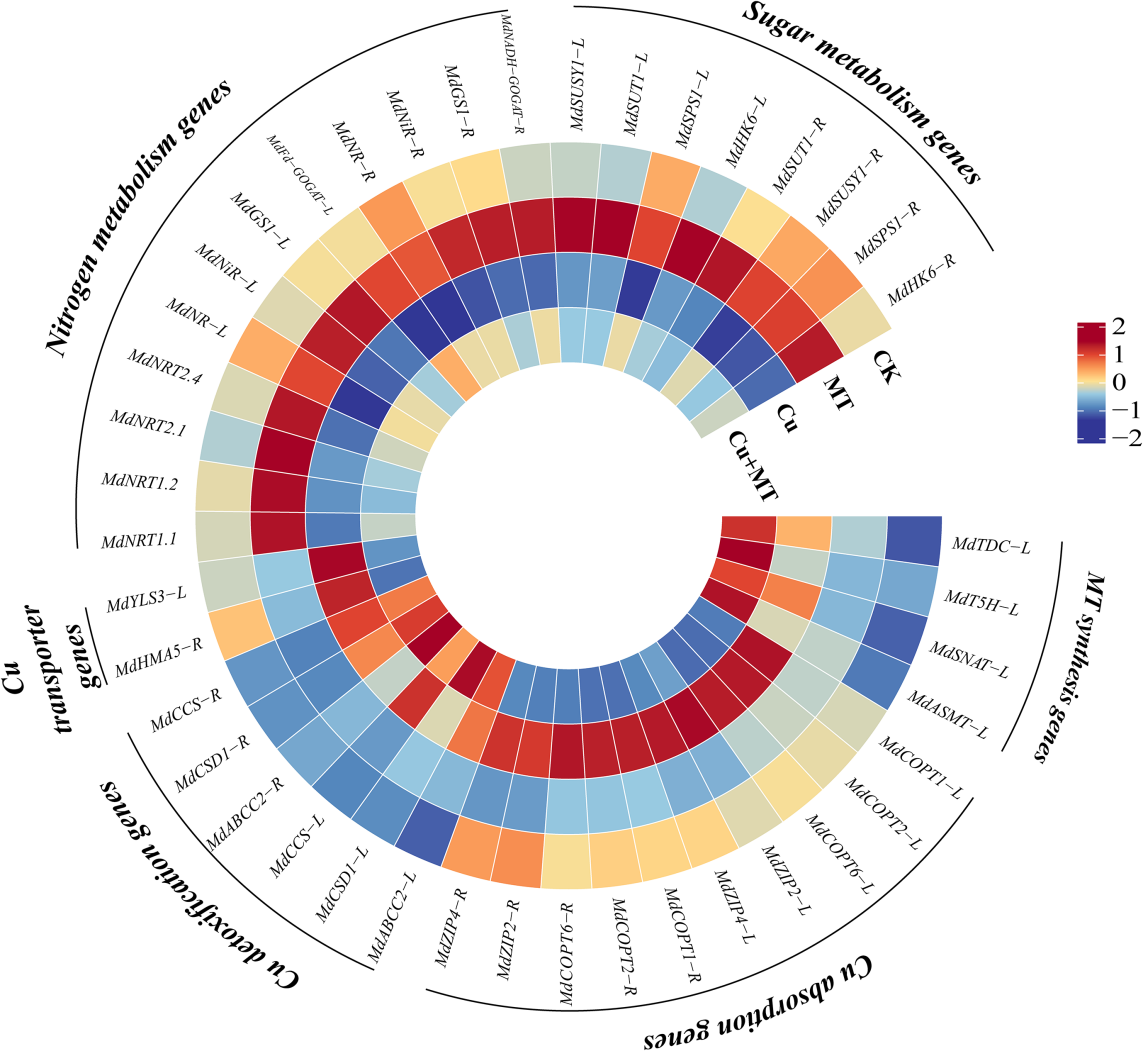
Expression of genes related to melatonin, copper absorption, transport, detoxification, and carbon-nitrogen metabolism in apple rootstock M9T337 seedlings under different treatments. “-L” represent leaves and “-R” represent roots. The color scale represents the relative intensity of gene expression. qRT-PCR results were standardized using the Z-score. Red squares indicate upregulation of gene expression, while blue squares indicate downregulation.

The effects of MT, Cu, and Cu+MT on the expression of MT synthesis-related genes in seedlings were significant (**Figure 9**). MT, Cu, and Cu+MT treatments upregulated the expression of leaf MT synthesis-related genes (*MdTDC*, *MdT5H*, *MdSNAT*, and *MdASMT*) compared to CK. Compared to CK, *MdTDC* expression in MT and Cu treatments increased by 2.12- and 3.03-fold, respectively. *MdTDC* expression was 1.36-fold higher in Cu+MT treatment than in Cu treatment.

To explore the molecular mechanisms underlying Cu absorption, transport, and detoxification in apple seedlings under different treatments, the expression of key genes involved in Cu absorption, transport, and detoxification in roots and leaves was analyzed (**Figure 9**). The study found that Cu stress increased the expression of copper absorption (*MdCOPT1*, *MdCOPT2*, *MdCOPT6*, *MdZIP2*, and *MdZIP4*), transport (*MdYSL3* and *HMA5*), and detoxification (*MdABCC2*, *MdCSD1*, and *MdCCS*) genes. Compared to Cu treatment, the addition of MT (Cu+MT treatment) could reduce the expression of Cu absorption and transport genes and increase the expression of Cu detoxification genes.

Under Cu stress, the expression levels of nitrate transporter genes (*MdNRT1.1*, *MdNRT1.2*, *MdNRT2.1*, and *MdNRT2.4*) in the roots of apple rootstock M9T337 and nitrogen assimilation-related genes (*MdNR*, *MdNiR*, *MdGS*, and *MdFd-GOGAT*) in leaves and roots were significantly downregulated (**Figure 9**). However, under Cu+MT treatment, the expression levels of these genes were significantly upregulated, indicating that MT effectively alleviates the inhibitory effect of Cu stress on the expression of genes related to nitrogen uptake and assimilation.

Compared to CK, sugar metabolism-related genes (*MdSUSY1*, *MdSUT1*, *MdSPS1*, and *MdHK6*) were upregulated under MT treatment, whereas they were downregulated under Cu treatment (**Figure 9**). The expression levels of sugar metabolism-related genes, such as *MdSUSY1*, *MdSUT1*, *MdSPS1*, and *MdHK6*, in the leaves increased under Cu+MT treatment compared to Cu treatment, suggesting that MT promotes carbon assimilation and transport under Cu stress by enhancing the expression of key sugar metabolism genes.

Therefore, Cu stress significantly inhibited the expression of nitrate transporter genes in the roots, nitrogen assimilation and reduction genes in the leaves, and carbon (sugar) synthesis-related genes; however, MT addition significantly alleviated this inhibitory effect and restored the expression of these genes to CK. Therefore, under Cu stress, MT enhanced nitrogen and carbon absorption and utilization by regulating the expression of genes related to carbon and nitrogen transport and metabolism, mitigating the negative impacts of Cu stress to some extent.

### 3.7 Correlation analysis

**Figure 10:**
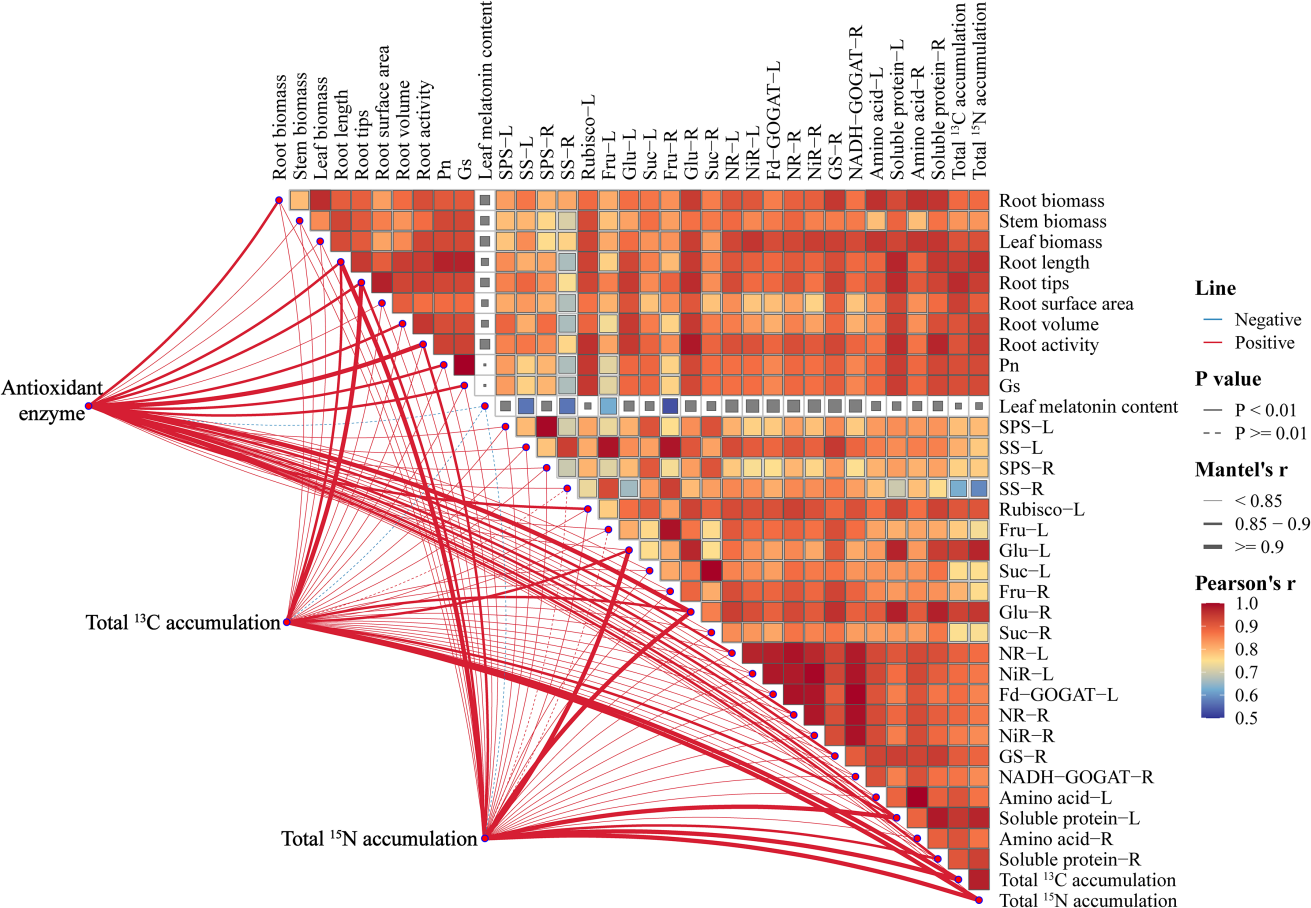
Correlation analysis between antioxidant enzymes, total ^15^N accumulation, total ^13^C accumulation, and physiological indicators. “-L” represent leaves and “-R” represent roots. Red line indicates positive correlation, and the thicker the line, the stronger the Mantel Test correlation. Color blocks represent Pearson correlation.

To determine the effects of different treatments on M9T337 seedlings, correlation analysis was performed on their growth indicators, carbon-nitrogen metabolism related indicators, antioxidant protective enzyme (SOD, POD, CAT, APX) activities, and the total accumulation of ^13^C and ^15^N. As shown in **Figure 10**, antioxidant enzyme activities were significantly positively correlated with root activity and Glu-R (Mental’s r > 0.9, *P* < 0.01). The total ^13^C accumulation was significantly positively correlated with root length, root tips, Glu content (leaves and roots), soluble protein, and total ^15^N accumulation (Mental’s r > 0.85, *P* < 0.01). There was a highly significant positive correlation between the total ^15^N accumulation and root length, root tips, root activity, Glu content (leaves and roots), and solule protein (leaves and roots) (Mental’s r > 0.85, *P* < 0.01).

## 4 Discussion

### 4.1 Melatonin alleviates copper stress inhibition of apple rootstock growth by improving root function

As the primary organ for plants to perceive environmental stress, the morphology and activity of roots directly determine the plant’s ability to absorb nutrients and water. A study has shown that excess Cu alters root morphology, thereby affecting the absorption of essential nutrients in rapeseed, consequently reducing growth, biomass, photosynthetic pigments, and the gas exchange capacity (Habiba et al., 2015). Cu exposure also induces severe oxidative stress, reducing biomass and growth in mustard seedlings (Yadav et al., 2023). Li et al. (2022) and Shi et al. (2021) found that MT alleviates Cu stress damage in wheat seedlings. The results of this study showed that the root length, number of root tips, root surface area, root volume, and root activity of M9T337 seedlings significantly decreased under Cu stress. Cu+MT treatment increased root system characteristics by 17.21−24.07% compared to Cu treatment, with synchronized recovery of root activity. MT may reduce Cu ion accumulation and toxicity in the roots by regulating the expression of cell wall synthesis-related genes and simultaneously promoting auxin synthesis and transport, which stimulates lateral root formation and improves root architecture (Wan et al., 2018; Arnao and Hernández-Ruiz, 2019).

### 4.2 Melatonin alleviates copper stress-induced oxidative damage by enhancing the antioxidant system to scavenge reactive oxygen species

Cu stress induces ROS accumulation in plants, leading to cell membrane lipid peroxidation and enzyme protein inactivation (Wang et al., 2024). In this study, the content of H_2_O_2_ and MDA in leaves and roots significantly increased under Cu treatment, while MT treatment alleviated oxidative damage through a dual mechanism: directly acting as an antioxidant to scavenge ROS and indirectly by upregulating the expression of antioxidant enzyme genes (e.g., SOD and POD), increasing enzyme activities. Cu+MT treatment significantly enhanced antioxidant enzyme activities compared to Cu treatment, consistent with the findings of Younis et al. (2018). The leaf MT content under Cu treatment increased by 23.76% compared to CK, which may be a self-protection mechanism of the plant in response to stress, consistent with the results of Huang et al. (2023) and Byeon and Back (2014). Exogenous MT application under Cu stress further increased the leaf MT content to 1.86 times that of CK, which resulted in a synergistic effect of “endogenous induction + exogenous supplementation, “ thereby more efficiently suppressing ROS accumulation. This provides evidence for elucidating the “dual regulatory role” of MT in plant stress resistance.

### 4.3 Melatonin optimizes carbon assimilation and product allocation by regulating photosynthetic efficiency and key enzyme/gene expression in carbon metabolism

Photosynthesis is the core of plant carbon metabolism, and inhibition of the photosynthetic system by Cu stress causes disorders in carbon metabolism. High Cu^2+^ concentrations induce lipid peroxidation in photosynthetic biomembranes, reduce the photosynthetic pigment content, gas exchange parameters, and chlorophyll fluorescence parameters in plants, inhibit photosynthesis, and increase cell wall lignification, thereby inhibiting plant growth (Zhao et al., 2018; Li et al., 2023). In this study, the leaf P_n_ and rubisco activity significantly decreased under Cu treatment, but MT application increased them, consistent with the findings of Ahmad et al. (2021) and Lin et al. (2022), who showed that MT improves plant photosynthetic efficiency. Downstream of carbon metabolism, Cu+MT treatment increased SPS and SS activities by 9.63 and 39.72%, respectively, compared to Cu treatment, with significant Fru and Glu accumulation and upregulation of genes, such as *MdSUSY1* and *MdSUT1*. This indicates that MT promotes sugar synthesis and transport at both the “enzyme activity” and “gene expression” levels. Furthermore, ^13^C isotope analysis showed that MT promotes the allocation of photosynthetic products to the roots, as the root ^13^C distribution rate under Cu+MT treatment increased by 16.25% compared to Cu treatment. Enhancing the carbon supply to the roots promotes root repair and growth and provides energy for the root absorption of mineral elements.

The enhancement of carbon metabolism not only provides the carbon skeletons and energy required for plant growth but also supplies critical precursors essential for the smooth progression of nitrogen metabolism. A central link between carbon and nitrogen metabolism lies in the provision of 2-oxoglutarate (2-OG), a key intermediate of the tricarboxylic acid (TCA) cycle. As an indispensable substrate for the GOGAT-catalyzed reaction in nitrogen assimilation, 2-OG accepts the amide group from glutamine to synthesize glutamate (Hodges, 2002). Our data demonstrated that MT treatment simultaneously significantly elevated GOGAT activity (**Figure 7**). Therefore, a plausible inference is that MT increased the carbon flux into the TCA cycle by promoting photosynthetic carbon fixation and sugar metabolism, thereby ensuring the sufficient production of 2-OG (Yang et al., 2022). This process furnishes an adequate carbon acceptor for the GOGAT-mediated nitrogen assimilation reaction, representing a potential regulatory node through which MT coordinately promotes carbon and nitrogen metabolism.

### 4.4 Melatonin maintains nitrogen metabolic balance under copper stress by improving the nitrogen uptake and assimilation efficiency

Nitrogen is an essential element for plants to synthesize key substances, such as proteins and nucleic acids. The inhibition of nitrogen metabolism by Cu stress exacerbates plant growth disorders. Using NMT, this study found that Cu stress caused a shift from NO_3_^−^ uptake to efflux in the root tips of M9T337 seedlings, but MT application reversed this trend, restoring the NO_3_^−^ uptake rate to −13.13 pmol·cm^−2^·s^−1^. The recovery of NO_3_^−^ uptake indicated that MT restored the integrity of root cell membranes and reestablished ion homeostasis under copper stress. This may be related to the upregulation of nitrate transporter genes, such as *MdNRT1.1* and *MdNRT2.1*, by MT. The transporters encoded by these genes are key carriers for root NO_3_^−^ uptake, and increasing their expression can enhance the root’s affinity and transport efficiency for NO_3_^−^. Compared to Cu treatment, Cu+MT treatment significantly increased the activities of NR, NiR, GS, and GOGAT in the nitrogen assimilation process and increased the amino acid and soluble protein contents by 21.33 and 18.45%, respectively, consistent with the results of Cao et al. (2022). Additionally, ^15^N isotope analysis showed that MT improves the nitrogen absorption and utilization efficiency, as the ^15^N utilization rate under Cu+MT treatment was 14.21% higher than that under Cu treatment. It also preferentially allocates nitrogen to the roots. This trend synergizes with the “allocation of photosynthetic products to the roots” in carbon metabolism, demonstrating that MT coordinates carbon and nitrogen allocation to provide material and energy support for root repair, which forms a regulatory network of “carbon–nitrogen interaction for stress resistance.”

Notably, vigorous nitrogen metabolism conversely regulates carbon metabolism. Nitrate reduction and assimilation consume substantial amounts of reducing power (NAD(P)H) and ATP generated by photosynthesis, while the synchronous increase in photosynthetic rate under melatonin treatment (**Figure 1j**) precisely meets this elevated energy requirement. Furthermore, 2-oxoglutarate (2-OG), the substrate for GOGAT (a key enzyme in nitrogen assimilation), is exclusively derived from carbon metabolism. Melatonin treatment significantly increased the contents of glucose and fructose (**Figure 4b**), providing an abundant carbon source for 2-OG biosynthesis. Additionally, glyceraldehyde-3-phosphate (G3P), an intermediate of glycolysis, serves as a carbon skeleton precursor for amino acids such as serine and glycine, which further participate in the photorespiratory cycle and nitrogen reassimilation (Igamberdiev and Kleczkowski, 2018). We hypothesize that while alleviating copper stress, melatonin may reprogram carbon metabolic flux to ensure the allocation of key intermediates (e.g., G3P and 2-OG) to nitrogen assimilation and amino acid synthesis pathways. This also explains the synergistic increase in soluble sugar, soluble protein, and amino acid contents observed under melatonin treatment (**Figures 4b**, **7**).

### 4.5 Melatonin achieves systemic alleviation of copper stress through multi-gene synergistic regulation

Gene expression analysis indicated that the alleviating effect of MT on Cu stress is the result of the synergistic action of multiple genes. In this study, exogenous MT upregulated the expression of MT synthesis genes, such as *MdTDC*, *MdT5H*, *MdSNAT*, and *MdASMT* (**Figure 9**), which is consistent with studies on copper stress in tomato (*Solanum lycopersicum*) and nutrient stress in apple rootstock (*Malus hupehensis*) (Zhang et al., 2022; Liu et al., 2023). We speculate that exogenously applied MT may not only act as a direct free radical scavenger but also be perceived by cells as a robust stress signal. Studies have shown that under copper stress, exogenous MT may activate downstream transcription factors (such as WRKYs, MYBs, NACs, etc.) through a signaling pathway mediated by melatonin receptors (e.g., PMTR1), which in turn bind to the promoters of endogenous MT synthesis genes to promote their expression (Wei et al., 2018; Arnao and Hernández-Ruiz, 2019). The present study also observed that under copper stress, the endogenous MT content had already increased, and the application of exogenous MT further elevated the MT content in leaves to 1.86 times that of the control, resulting in a synergistic effect of “endogenous induction + exogenous supplementation” and more effectively inhibiting ROS accumulation.

MT down-regulated the expression of copper absorption (such as *MdCOPT1* and *MdCOPT2*) and transport genes (*MdYSL3* and *HMA5*), and up-regulated the expression of copper detoxification genes (*MdABCC2*, *MdCSD1*, and *MdCCS*), thereby reducing the copper content in seedlings. YSL3 has been confirmed to be involved in the xylem loading process of metal ions from roots to aboveground tissues (Sheng et al., 2021). In this study, the expression of *MdYSL3* was downregulated under the Cu+MT treatment (**Figure 9**), accompanied by a significant decrease in leaf copper content (**Figure 3d**), indicating that melatonin limits copper translocation to leaves by downregulating *MdYSL3*, thereby protecting photosynthetic organs from copper toxicity. This is consistent with the observations in the present study that melatonin treatment increased the leaf P_n_ and rubisco activity (**Figures 1j**, **4a**), demonstrating that reducing copper accumulation in leaves is an important mechanism for maintaining photosynthetic function. MT synchronously regulated the expression of carbon metabolism-related genes (*MdSUSY1* and *MdSPS1*) and nitrogen metabolism-related genes (such as *MdNR* and *MdNRT1.2*), shifting the expression of carbon and nitrogen metabolism-related genes from significantly downregulated under Cu stress to upregulated expression. This multi-gene synergistic regulation mode breaks the suppression of carbon and nitrogen metabolism gene expression under Cu stress, achieving the systemic recovery of plant physiological functions. This provides a basis for analyzing the mechanism by which MT alleviates heavy metal stress in plants at the molecular level.

Therefore, the role of melatonin in alleviating copper stress-induced inhibition of carbon and nitrogen metabolism is a dual process: on the one hand, it directly acts as a signaling molecule, upregulating the expression of genes related to carbon and nitrogen metabolism, enhancing the activity of key enzymes, and promoting carbon and nitrogen assimilation and accumulation, thereby actively coordinating and enhancing the overall metabolic function of plants to cope with stress; on the other hand, it indirectly reduces the toxicity of heavy metals to metabolic enzymes and organelles by downregulating genes such as *MdCOPT2* and *MdYSL3*, thereby decreasing copper absorption and its transport to aboveground tissues; these two mechanisms work synergistically to achieve efficient recovery and balance of carbon and nitrogen metabolism under copper stress.

## 5 Conclusion

**Figure 11:**
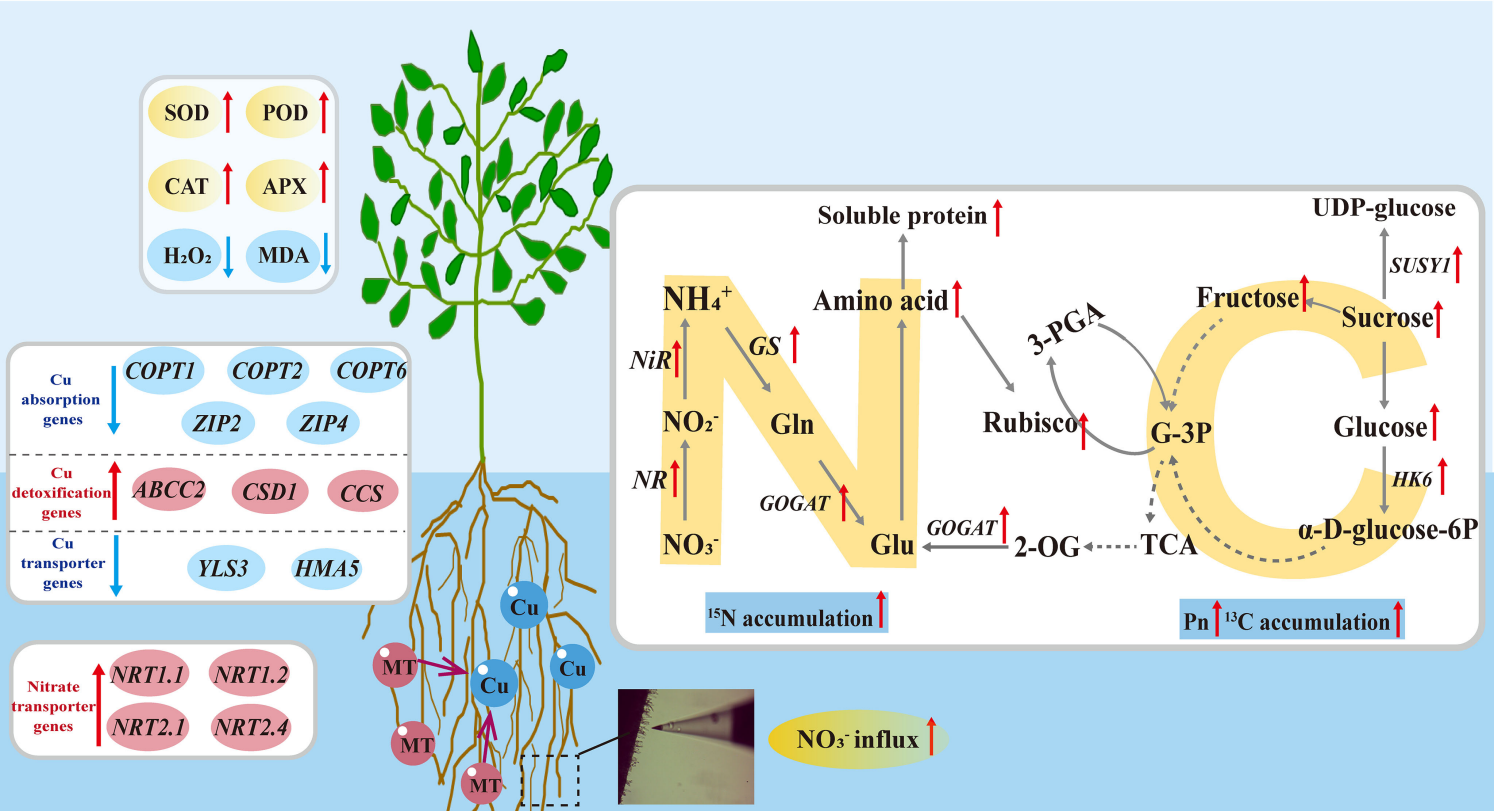
A model of the effect of exogenous melatonin on the antioxidant system and carbon-nitrogen metabolism of apple rootstock under copper stress.

Copper stress induced the accumulation of ROS in apple rootstock seedlings, resulting in oxidative damage and disruption of carbon-nitrogen metabolism. Exogenous melatonin enhanced antioxidant enzyme activities, reduced the content of H_2_O_2_ and MDA, mitigated oxidative damage, and supported normal plant growth. Under copper stress, the application of melatonin reduced the expression of copper absorption and transport genes, while enhancing the expression of copper detoxification genes. Simultaneously, melatonin application promoted nitrate influx in root tip, upregulated the expression of root nitrate transporter genes (*MdNRTs*), improved nitrogen assimilation in leaves and roots, and increased ^15^N accumulation. Concurrently, melatonin elevated P_n_, soluble sugar content, and ^13^C accumulation (**Figure 11**). Therefore, exogenous MT significantly alleviated the negative effects of Cu stress on apple rootstock M9T337 by enhancing its antioxidant capacity, optimizing the accumulatio and allocation of photosynthetic products, and improving nitrogen uptake and utilization. These findings provide a theoretical basis for managing soil Cu pollution in apple orchards and improving apple production quality and efficiency. Future research should further explore the synergistic effects of MT with other plant hormones and the genotypic differences in the response of apple rootstocks to MT, laying the foundation for precise MT application in the apple industry.

## Data Availability

The original data presented in the study are included in the article/**Supplementary Material**. Further inquiries can be directed to the corresponding authors.
