# Supplementary material for: Exogenous melatonin alleviates copper stress in apple rootstock M9T337 by regulating the antioxidant system and carbon–nitrogen metabolism
Source: Front Plant Sci. 2026 Mar 18;17:1793846. doi: 10.3389/fpls.2026.1793846 (PMC13038588; doi:10.3389/fpls.2026.1793846)
Supplement: Supplementary file 1 [file Table1.docx]

**Table S1** Primer sequences for qRT-PCR.

| **Gene**  **name** | **Forward sequence of the primers**  **(5′→3′)** | **Reverse sequence of the primers**  **(5′→3′)** |
| --- | --- | --- |
| *MdNRT1.1* | CTCGGCCTCATTGTGTTCTT | TCCAACGGCAGTTCCATATTC |
| *MdNRT1.2* | TTAATTGCTGCCACACTTCATAG | CACGATGTTTGGTTCTGATACTTC |
| *MdNRT2.1* | GCTGTACTCTTCCTGTGACTTT | CGTCGACTTCTCGACATCTTT |
| *MdNRT2.4* | CAGAAGGTGAACCCGGAAG | CAAGTGGAACGTCCTCATGTG |
| *MdNR* | GTCACACGAGTGGAGATAACAA | CAGAAACACCAGCACCAGTA |
| *MdGS1* | ATATCTGCTGGAGATGAACTGTGG | TGGACTTGGTGCTGTAGTTTGTG |
| *MdFd-GOGAT* | CGAAGGAAGAAGAAGACCACGC | TTGCTGGTGCCTGTTGGGTT |
| *MdNADH-GOGAT* | ACTATGGTCGGTTCTCAAC | TCTTGATGCCTCTTGCTAA |
| *MdSUSY1* | CTC AAG CGT GTT AAG CAA CAG | CTG AAT GGA ACA CGA AGA ATA TC |
| *MdSUT1* | TGT TCC GTA TGC TTT GGT TTC TTC | AAT AGC TGA TCC CAA GGT CCA CT |
| *MdSPS1* | AGT GTA GTA CTC AAG GGA GTT GG | TGC TCA TGG GGA AGG CTT TAC |
| *MdHK6* | GTG GGG CAG AGT GTT TGG TGT T | AAC CAC CGT CAG AGG CCA AAC C |
| *MdTDC* | TCACGCTGTGGTTGGAGGT | CTGCATGCTCCTGAACCAAC |
| *MdT5H* | TCGGTGACATGTTTGCTGC | GGAAACCTTGGTCTGGCG |
| *MdSNAT* | GAATCACCGTCCACGCTCC | GAAATGCTTCCGATGTCCC |
| *MdASMT* | AGAGGAGCGAGAAAGACTCGA | CTAAAGAAAAACTTCAATGAGGGAT |
| *MdCOPT1* | ATGACGAACGGTACGACGAT | AGGGCAAAGACAAAGAATAAG |
| *MdCOPT2* | CATGACGAACGGCACGAT | GCCAACCCAACCCTAACTG |
| *MdCOPT6* | CTCGGCATGTACGTCTTG | GGAGGGATAGGTAGGGTTT |
| *MdZIP2* | CTAATAGATGCGACAACCC | GCCACATAAGCCCAATAC |
| *MdZIP4* | CTGTCGCTTCGATTCTCCT | CCCAAACACCTTCCCATT |
| *MdABCC2* | ACGTTGGGACTGATGCTCTT | TGTCTGGGGAGTCATGTTCC |
| *MdCCS* | CGTTAGGGTTCTGGGTTCAAC | CGAACCACACCAAACACATC |
| *MdCSD1* | AATCCTGCTGGAAAAGAGCA | CCAGCATTTCCTGTGGATTT |
| *MdYSL3* | GCGAAAACGGAGAGATTGAG | AGAAGTGCAGCCGAAACATTG |
| *MdHMA5* | AATTGGAAAGCCAGTGGTTG | GGCCTTAGCCAACGGGTGTTC |
| *MdActin* | CATGGTTGGTATGGGTCAGAAG | GTCATCCCAGTTGCTCACTATG |
